# Clinical utility of lymphocyte transformation testing for antituberculosis drug hypersensitivity

**DOI:** 10.1016/j.jdin.2026.09.011

**Published:** 2026-10-05

**Authors:** Patchara Pornsuthirat, Tunsuda Tansit, Somkiat Ud-naen, Papapit Tuchinda, Nasikarn Angkasekwinai, Nitipatana Chierakul, Ticha Rerkpattanapipat, Jettanong Klaewsongkram, Yuttana Srinoulprasert

**Affiliations:** a Department of Immunology, Faculty of Medicine Siriraj Hospital, Mahidol University, Bangkok, Thailand; b Department of Dermatology, Faculty of Medicine Siriraj Hospital, Mahidol University, Bangkok, Thailand; c Department of Medicine, Faculty of Medicine Siriraj Hospital, Mahidol University, Bangkok, Thailand; d Faculty of Medicine Ramathibodi Hospital, Mahidol University, Bangkok, Thailand; e Division of Allergy and Clinical Immunology, Department of Internal Medicine, Center of Excellence for Skin and Allergy Research, Faculty of Medicine, Chulalongkorn University, Bangkok, Thailand

**Keywords:** antituberculosis drugs (ATDs), drug allergy, lymphocyte transformation test (LTT), multiple drug allergy (MDA)

*To the Editor:* Severe cutaneous adverse reactions and maculopapular exanthema (MPE) induced by antituberculosis drugs (ATDs) pose a significant challenge for dermatologists, particularly in cases of multiple drug allergy (MDA). While in vivo drug provocation testing (DPT) is the diagnostic gold standard, it carries an unacceptably high risk of triggering life-threatening cutaneous flare-ups in severe delayed allergic reactions. We evaluated the in vitro lymphocyte transformation test (LTT) to safely identify culprit ATDs.

This study comprised a validation cohort (20 confirmed ATD allergies, 15 ATD nonallergies, 9 healthy controls) and a retrospective chart review of 15 ATD-allergic patients. Dermatologists clinically confirmed diagnoses using drug-exposure timeline and causality tools. Detailed methods are provided in the Supplementary Materials (available via Mendeley at https://data.mendeley.com/datasets/ws8js89f2m/1). At our institute, patch testing is routinely performed for contact dermatitis but lacks protocols for systemic drug allergy. Furthermore, DPT slots are limited and ethically contraindicated in severe cutaneous adverse reactions because of life-threatening risks. Instead, clinicians safely reintroduced LTT-negative drugs, supporting causality assessments. LTT positivity was defined as stimulation index >2 at any of the 3 tested nontoxic concentrations.^1^

Since DPT with suspected culprits is contraindicated in severe cutaneous adverse reactions, diagnostic sensitivity is difficult to establish. Therefore, performance was evaluated as a positivity rate of 60.0%, consistent with previous reports.^2^, ^3^ The LTT demonstrated a true specificity of 95.8% against the clinically defined control groups (Table II). Crucially, reintroduction findings in 15 patients served as supportive clinical follow-up: safe reintroduction of selected LTT-negative ATDs in 12 of these patients (MPE and DRESS) resulted in no cutaneous flare-ups, supporting the assay’s clinical utility.

In the retrospective cohort, 73.3% had at least 1 positive LTT result. Isoniazid (58.3%) and rifampicin (64.3%) were the most frequent culprit drugs (Table I).^4^ Notably, 63.6% of LTT-positive patients exhibited MDA (Table II). Several factors influence LTT positivity rates and stimulation index magnitude, including time elapsed since the initial reaction, type of allergic reaction, and assay methodology. This variability was evident, as 7 patients exhibited only weakly positive signals (stimulation index, 2.0-3.0) (Supplementary Table 3, available via Mendeley at https://data.mendeley.com/datasets/ws8js89f2m/1). Despite being weak, these signals remain clinically informative given the assay’s high specificity, emphasizing the necessity for careful interpretation alongside the patient’s clinical history.

**Table I:** Characteristics of volunteers in validation cohort and retrospective cohort

	Healthy controls	ATD nonallergy	ATD allergy	Retrospective cohort
Number of volunteers	9	15	20	15
Median (y)	24	35	63.5	63.0
Gender (male/female)	6/3	4/11	4/16	4/11
Number of positive/number of tests				
Isoniazid	0/9	0/15	6/19	7/12
Rifampicin	0/9	1/15	6/20	9/14
Pyrazinamide	0/9	0/15	2/16	2/12
Ethambutol	0/9	0/15	4/18	1/12
Types of drug allergy symptoms				
MPE	NA	NA	11	7
DRESS	NA	NA	6	7
SJS/TEN	NA	NA	1	1
Erythema multiforme	NA	NA	2	0

The number of tests might not equal the number of volunteers in each group if not all drugs were tested for each individual. *DRESS*, drug reaction with eosinophilia and systemic symptoms; *MPE*, maculopapular exanthema; *NA*, not applicable; *SJS*, Stevens-Johnson syndrome; *TEN*, toxic epidermal necrolysis.

**Table II:** LTT performance

	Healthy controls	ATD nonallergy	ATD allergy	Retrospective cohort
Number of positive/number of tests				
Isoniazid	0/9	0/15	6/19	7/12
Rifampicin	0/9	1/15	6/20	9/14
Pyrazinamide	0/9	0/15	2/17	2/12
Ethambutol	0/9	0/15	4/19	1/12
LTT results				
Positive rate	0/9	1/15	12/20	11/15
Negative rate	9/9	14/15	8/20	4/15
MDA (≥2 positive drugs)	0/9	0/15	5/20	7/11

*MDA*, multiple drug allergy.

A major limitation is the lack of direct DPT comparison across all subjects, which is ethically unfeasible in these severe dermatologic cohorts. Regarding age and gender, older patients theoretically possess altered baseline immune activation that could yield false-positive in vitro results. Subgroup analysis of our validation allergy cohort revealed no significant difference in LTT positivity between patients >60 years (61.5%) and ≤60 years (57.1%), suggesting that age does not drive hyperreactivity. The gender-demographic disparity between ATD-allergy and control cohorts necessitates acknowledgment. Larger prospective studies with age- and gender-matched patient populations are needed to further validate these findings. Furthermore, the high prevalence of MDA (63.6%) in the retrospective cohort aligned with dermatologic literature, where massive immune activation during intense systemic reactions like DRESS lowers the threshold for T-cell neo-sensitization.^5^ Despite a moderate positivity rate, the LTT’s high specificity—corroborated by safe clinical reintroductions—makes it a valuable adjunctive tool for dermatologists navigating complex cutaneous ATD allergies.^1^

### Declaration of generative AI and AI-assisted technologies in the writing process

The authors used Gemini 2.5 Pro and Grammarly Pro, AI-assisted editing tools, to improve spelling, grammar, clarity, and readability during manuscript preparation. After using this tool, the authors carefully reviewed and edited the text and took full responsibility for the final content.

## Conflicts of interest

None disclosed.
